# Finding and Characterizing the Complexes of Drug Like Molecules with Quadruplex DNA: Combined Use of an Enhanced Hydroxyl Radical Cleavage Protocol and NMR

**DOI:** 10.1371/journal.pone.0096218

**Published:** 2014-04-24

**Authors:** Harikrushan Ranpura, Dobroslawa Bialonska, Philip H. Bolton

**Affiliations:** Chemistry Department, Wesleyan University, Middletown, Connecticut, United States of America; University of Quebect at Trois-Rivieres, Canada

## Abstract

Structural information on the complexes of drug like molecules with quadruplex DNAs can aid the development of therapeutics and research tools that selectively target specific quadruplex DNAs. Screening can identify candidate molecules that require additional evaluation. An enhanced hydroxyl radical cleavage protocol is demonstrated that can efficiently provide structural information on the complexes of the candidate molecules with quadruplex DNA. NMR methods have been used to offer additional structural information about the complexes as well as validate the results of the hydroxyl radical approach. This multi-step protocol has been demonstrated on complexes of the chair type quadruplex formed by the thrombin binding aptamer, d(GGTTGGTGTGGTTGG). The hydroxyl radical results indicate that NSC 176319, Cain’s quinolinium that was found by screening, exhibits selective binding to the two TT loops. The NMR results are consistent with selective disruption of the hydrogen bonding between T4 and T13 as well as unstacking of these residues from the bottom quartet. Thus, the combination of screening, hydroxyl radical footprinting and NMR can find new molecules that selectively bind to quadruplex DNAs as well as provide structural information about their complexes.

## Introduction

Quadruplex DNAs have been implicated in a number of biological processes.[1]–[7] Quadruplex DNA has been proposed to be important in telomere structure and function, in the regulation of gene expression and in some triplet repeat diseases.[2], [5], [6], [8]–[11] The importance of these biological roles and the variety of quadruplex structures has given rise to interest in finding drug like molecules that bind to specific quadruplex DNAs for use as potential leads for therapeutic intervention and as research tools to investigate the presence and functions of quadruplex DNA in cells and other complex systems such as shelterin.[1]–[4], [6]–[8], [11]–[16] The structures of the complexes of quadruplex DNAs are also of interest in the development of sensors based on aptamers that contain quadruplex structures [17].

Moderate to high throughput screening of drug like molecules against a target can be a first step in identifying potentially useful candidates. Initial screening is typically designed to find all, or almost all, candidate molecules that are then subject to further scrutiny. Quite a number of methods have been developed that screen molecules for their binding to quadruplex DNAs.[9], [11], [18]–[20] We have designed a screening method that aims to identify molecules that preferentially bind to quadruplex structural types.[9] Methods have been developed to screen for accurate monitoring of telomerase inhibition [21] and for the presence of quadruplex DNA *in vivo*.[22] The current screening methods do not distinguish between binding to a single or to many sites of the quadruplex DNA.

There is a need for an efficient protocol that can provide structural information about the complexes of quadruplex DNAs identified by screening as well as differentiate between molecules that bind to a single site from those that bind to multiple sites. Nucleotide resolution structural information can be sufficient for this purpose. We have developed an enhanced hydroxyl radical cleavage protocol that provides nucleotide resolution structural information about the complexes of drug like molecules with quadruplex DNAs. This approach is usable over a range of temperature and counterion, DNA and drug like molecule concentrations. This protocol can also be used to examine the binding constants, stoichiometry and other properties of the complexes. For example, the investigation of the extent of cleavage as a function of the concentration of the drug like molecule could allow determination of the binding constant of the complex [23], [24].

The results of the hydroxyl radical cleavage approach can be extended and validated by investigation of the complexes by NMR methods. The combined use of screening, hydroxyl radical footprinting and NMR has allowed the identification and characterization of the selective complex formed by Cain’s quinolinium [25], [26], NSC 176319, with quadruplex DNA.

The structures of quadruplex DNAs with a number of different folding patterns have been determined and a considerable diversity in the loops that connect the quartets has been observed.[1], [2], [4], [6], [7], [10], [27]–[31] These structures also exhibit a range of groove widths and electrostatic potentials.[32] While the range of structural features for quadruplex DNA may not be as great as has been observed for proteins or RNA there is enough structural diversity [33] to suggest that quadruplex DNAs may well have regions that allow selective targeting of particular quadruplex structural types.[1] Crystallography and NMR have been used to determine the structures of quadruplex DNAs and their complexes. [1], [2], [4], [6], [7], [10], [27]–[31], [34] The cleavage of quadruplex DNAs has been used to investigate the binding sites of radical generating molecules [35], [36].

**Figure 1:**
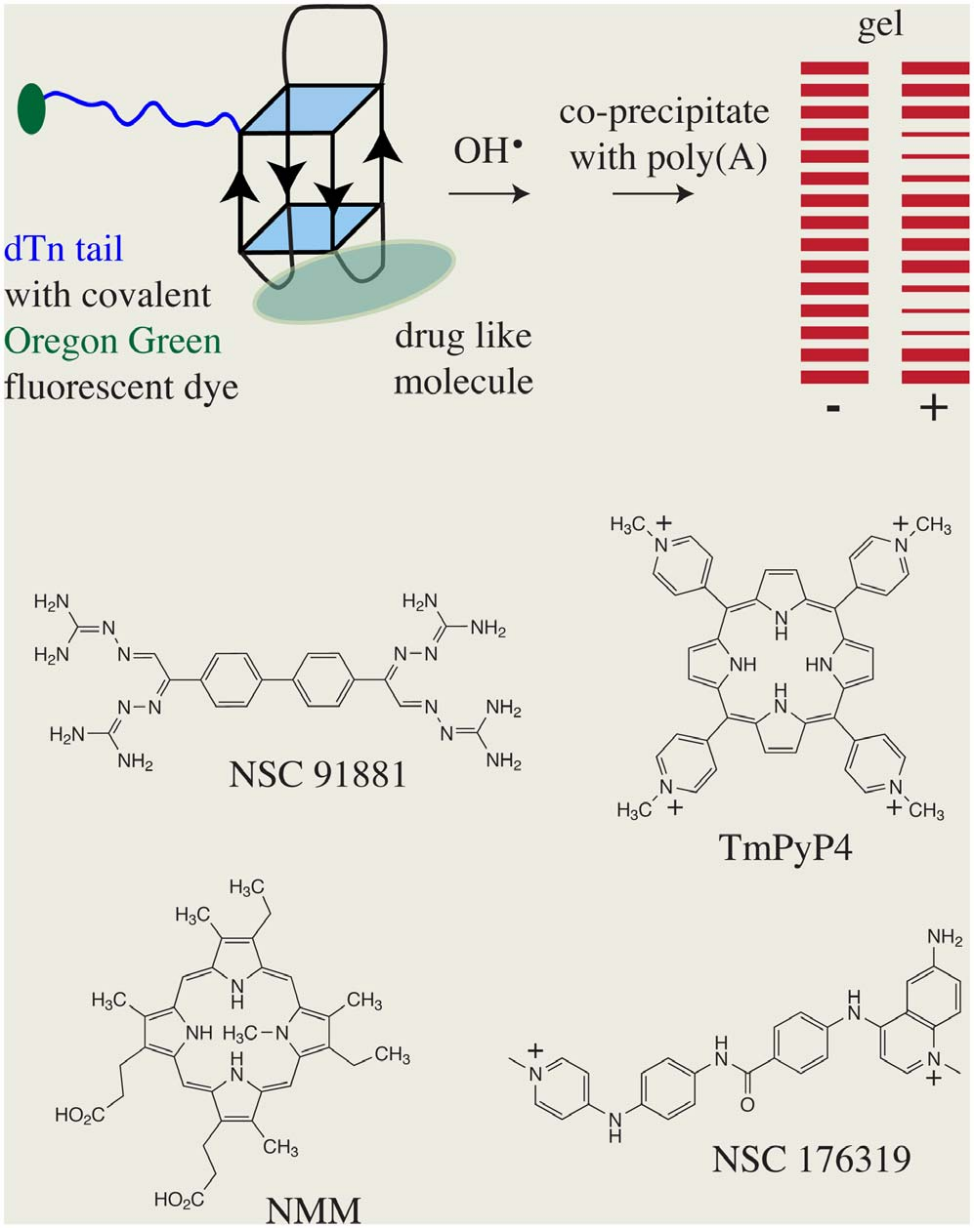
The overall scheme for the hydroxyl radical cleavage approach is depicted. The quadruplex DNA has a dT_n_ tail, depicted in blue, that is covalently attached to a fluorescent Oregon green, depicted in green. The DNA is reacted with hydroxyl radical in the presence and absence of a drug like molecule and the fragments separated by gel electrophoresis. The intensities of the bands are compared to gain information about the location of the binding sites of the drug like molecule. The structures of the four drug like molecules used in this investigation are also shown.

Screening has been able to identify a number of potentially interesting molecules that bind to quadruplex DNAs.[9], [11], [18], [19], [21], [37] Efficient methods for obtaining structural information on the complexes are needed for critical evaluation of the candidate molecules. An enhanced hydroxyl radical cleavage protocol can provide nucleotide resolution structural information about the using complexes of drug like molecules. This approach is demonstrated here with the chair type quadruplex structure formed by the 15 mer d(GGTTGGTGTGGTTGG) that is often referred to as the thrombin binding aptamer, TBA. [31], [32], [38]–[40] An overview of the protocol is depicted in Figure 1. The extent of hydroxyl radical cleavage at a particular residue is proportional to the solvent accessibility of the sugar of that residue.[41]–[44] Formation of a complex can also lead to enhanced cleavage by altering the structure such that the sugar is more exposed to hydroxyl radical [41].

The hydroxyl radical is a neutral molecule and this is an important feature as many quadruplex DNAs have large electrostatic potentials. It is noted that this approach also has the potential to gain new information about the folding patterns of quadruplex DNAs. The crystal structure of the ribosome was used to validate the application of the hydroxyl radical cleavage approach to the monitoring of complex nucleic acid folding patterns and interactions.[44], [45] The changes in extent of cleavage due to drug like molecule binding can indicate which residues of the DNA are spatially close to one another as depicted in Figure 1. This approach could also be applied to the quadruplex aptamers used in sensors [17].

The drug like molecules used here include NMM and TmPyP4 that have been previously identified as quadruplex binders.[8], [12], [19], [46]–[48] NSC 176319, Cain’s quinolinium was found using the screening method previously described.[9] NSC 91881 was identified in a structurally similarity search to NSC 176319 and then screened. The results of the hydroxyl radical approach have been validated and extended by the application of NMR methods to the complexes.

## Materials and Methods

### DNA Samples

DNA was obtained from Integrated DNA Technologies and Sigma in HPLC purified form. The DNAs were dissolved in 100 µL of 2.5 mM Na_2_HPO_4_, pH 7. These samples were then precipitated with 10 µL of 3 M NaCl and 500 µL of chilled ethanol. After three hours of incubation on ice the samples were centrifuged at 13000 rpm and supernatant was removed. The resulting pellet was dried using a speed-vac. The concentration of the samples was determined by absorption using a Nanodrop spectrometer.

### Oregon Green Labeling

The DNA samples with a modified amine, 5AmMC6T, at the end of the dT_11_ tail, were dissolved in 100 µL of autoclaved, doubly distilled H_2_O, extracted one time with an equal volume of phenol, then twice with chloroform and ethanol precipitated as above. The DNA was then dissolved in 100 µL of water at 100 µM to which 250 µg of Oregon Green 514 carboxylic acid succinimidyl ester, from Invitrogen, in 14 µL DMSO and 75 µL of 0.1 sodium tetraborate at pH 8.5 were added. The reaction mixture was incubated overnight on a shaker at low oscillation. The labeled DNA was purified by three ethanol precipitations to remove the free Oregon Green. The extent of labeling was estimated by comparing the absorption at 260 nm and that at 480 nm. The extent of labeling was found to be greater than 90% for all samples used in this study.

### DMS Footprinting

The reaction mixtures consisted of 1 µL of 0.1 M DMS and 20 µL of 2.5 µM Oregon Green labeled DNA. After 10 min incubation at room temperature 5 µL of 30 mM poly(A) was added and the DNA was precipitated with 100 µL chilled ethanol. After three hours on ice the samples were centrifuged at 13,000 rpm, the supernatant was removed and the pellet was dried using a speed vac.

### Pyrrolidine Treatment

The DNA samples were dissolved in 20 µL of 0.1 M pyrrolidine and heated at 363 K for 30 minutes. After cooling on ice the samples were dried using a speed-vac and lyophilized. The samples were dissolved in 30 µL of autoclaved water and lyophilized a second time to remove any residual pyrrolidine.

### Quadruplex Sample Preparation for Hydroxyl Radical Reaction

The labeled DNA was dissolved in 100 mM NaCl, 10 mM KCl and 2.5 mM Na_2_HPO_4,_ pH 7.0. The DNA samples were heated to 363 K, in a water bath, for 8 minutes and then allowed to cool to room temperature overnight.

### Hydroxyl Radical Cleavage Reaction

The hydroxyl radical cleavage reaction was initiated by adding one-twentieth volume each of the 10 mM Fe(II)/20 mM EDTA solution, 100 mM sodium ascorbate and 0.04% H_2_O_2_, 1∶800 dilution of a 30% solution, to the DNA sample. After 1 min the reaction was quenched by one-fourth volume of 230 mM thiourea. The H_2_O_2_ was a 1∶100 dilution of a 30% solution. The sample was then ethanol precipitated after addition of one-fourth volume of 30 mM poly(A). The samples were then subjected to the pyrrolidine treatment. The pyrrolidine treatment regenerates Oregon Green fluorescence lost by the hydroxyl radical reaction and removes oxidized deoxyribose fragments from the 3′ phosphate. The DNA was 2.5 µM in the hydroxyl radical reactions. The drug like molecules were added at 2.5, 5 and 10 µM before the cleavage reactions were begun.

### Gel Electrophoresis and Imaging

The DNA samples were dissolved in 5 µL of autoclaved water and 5 µL of ultrapure formamide. After heating to 363 K for 4 minutes and cooling on ice the samples were loaded on a gel that had been pre-run at 45 W for 40 min. The gels used are 20 cm×40 cm, 0.75 mm, 15% polyacrylamide denaturating gel in TBE buffer, 0.1 M Tris base, 0.1 M boric acid and 1 mM EDTA at pH 8. Electrophoresis was carried out at 45 W for 5 hours.

The gels were imaged using a Typhoon Trio with the fluorescence scanning at the green-excited mode at 532 nm with the emission filter at 526 nm and the photomultiplier at 600 V. The intensities of all of the bands were within the dynamic range of the imager.

The gel image was analyzed using Semi-Automated Footprinting Analysis software SAFA v1.1 [43] as described previously.[42] The results were exported to Excel. The sum of the intensities in each lane was normalized and all of the results shown are the average of three separate experiments. The percentage change in the normalized intensities of each individual band was calculated to generate the histograms presented below. An example of the normalization procedure is shown in File S3.

The reproducibility of the procedure was determined by running a cleavage reaction seven times. The analysis of the results indicated that the standard deviation in the intensity of any single band is on the order of 7% with the standard error of about 2% as described previously.[42] The results reported here are the averages of three runs.

### NMR Experiments

All of the NMR experiments were carried out using a Varian Inova 600 MHz spectrometer with the samples at 288+/−1 K. The 1 mM TBA samples were in 100 mM NaCl, 10 mM KCl and 10 mM phosphate buffer at pH 7. The samples were in 95% H_2_O and 5% ^2^H_2_O and ‘wet’ water suppression was used. The delay time between experiments was 3 s and the acquisition time was 0.256 s. The data was processed using Varian 6.1C software.

### Solvent Accessible Surface Area Calculations

The structures of the DNAs were 1C32 [49] and 148D [39] from the Protein Data Bank both of which were determined by NMR based methods. The water accessible surface areas of the sugar hydrogen atoms were calculated using Chimera 1.5.3rc software. The surface was computed using Molecular Surface MSMS. A render/select by attribute function was selected in the Model panel and a solvent accessible surface area of the radius 0.14 nm was calculated for individual atoms. The relative surface area accessibilities of the residues from the two structures are listed in File S1.

## Results and Discussion

The quadruplex DNAs of interest are typically much smaller than most of the DNA and RNA that have been investigated by hydroxyl radical cleavage reactions. One of the challenges with the use of a small DNA is the purification of the even smaller fragments from the reagents that are used to generate and to quench the hydroxyl radicals. The efficiency of ethanol precipitation is strongly dependent on the length of the fragments smaller than 30–40 nucleotides. The length dependence is a function of total salt and temperature and this can lead to low reproducibility with the small DNA fragments. These challenges can be overcome by the use of an enhanced hydroxyl protocol that was adapted from our prior work on duplex DNAs containing damaged sites and their complexes with drug like molecules [42].

The enhanced protocol uses poly(dT_11_) tails to increase the length of the smallest fragments along with poly(A) co-precipitation to increase the efficiency and uniformity of precipitation of fragments as depicted in Figure 1. A stable fluorescent dye, Oregon Green, is covalently attached to the end of the poly(dT_11_) tail giving high sensitivity detection of the fragments and the ability to store the labeled samples for long periods of time. The poly(dT_11_) tail can be on the 5′ or 3′ end.

**Figure 2:**
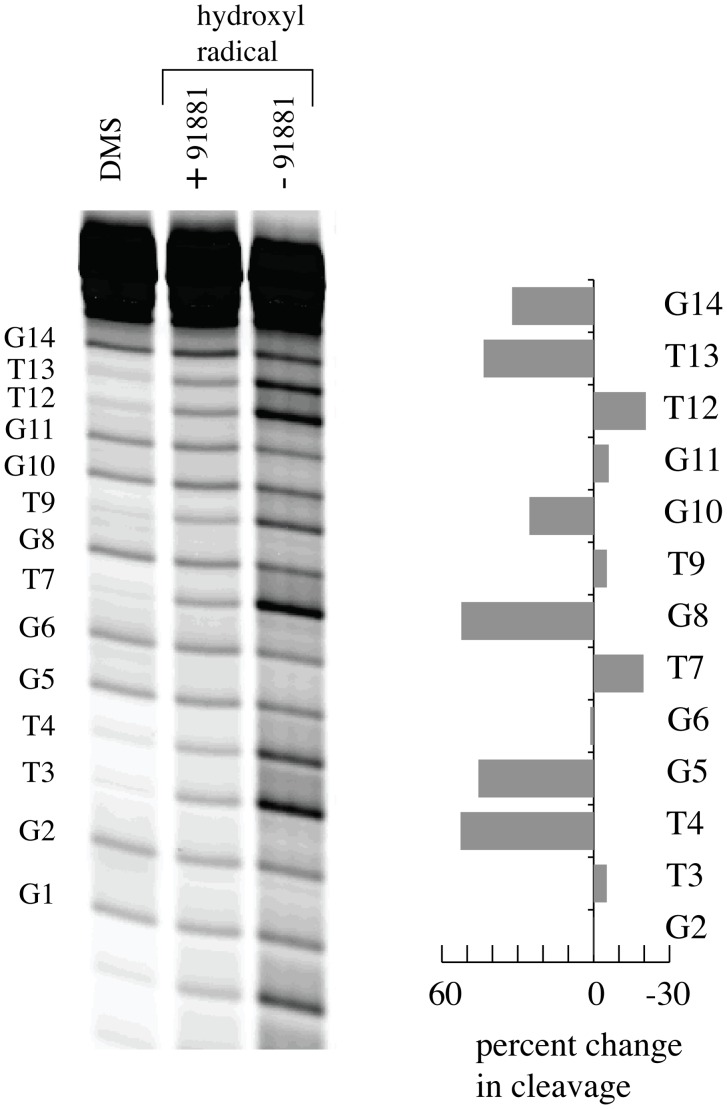
Representative cleavage results in the presence and absence of a drug like molecule. The cleavage results for the hydroxyl radical cleavage of the sample with the dT_11_ tail on the 5′ end in the presence and absence of NSC 91881 are shown along with the results of a DMS cleavage. The changes in cleavage due to the presence of NSC 91881 are depicted for each band in the histogram on the right.

A typical gel of the fragments generated by the hydroxyl radical cleavage of TBA in the presence and absence of a drug like molecule, in this case NSC 91881, is shown in Figure 2 along with a DMS cleavage lane. The percentage change in cleavage is presented as a histogram in Figure 2. The cleavage patterns for the samples with 5′ and 3′ tails give similar results and the cleavage results are tabulated in File S4. The examination of samples with 5′ and 3′ tails gives complementary information since the fragments that are near the parent band in the gels are the most challenging to observe. For example, the bands resulting from cleavage at G15 and G14 are near the parent band with the 5′ tail sample while G1 and G2 are with the 3′ tail sample. The residues with the highest level of cleavage are T3, T7 and T12 all of which are in loop regions.

Two of the NMR based structures of the 15 mer [39], [49] have been used to calculate the accessibility of the deoxyribose of the sugars. The surface accessibility calculations, results shown in File S1, predict that T3 and T12 will be cleaved more than the adjacent T4 and T13 as is observed. The quartet residues are predicted to have less cleavage than the loop residues in agreement with the experimental results. Both the predicted and the experimental results have G8, in the top loop, having relatively low reactivity.

The experimental cleavage results and the surface accessibility calculations generally agree about which residues are the most exposed. The accessibility calculations predict more relative protection for the residues in the quartets than is observed in the cleavage reactions. The accessibility calculations did not include tightly bound water molecules or cations. The inclusion of water and cations may bring the predictions closer to the observed results.

### The Presence of a Tail does not Change the CD Spectrum

This 15 mer has the characteristic CD spectrum of an anti-parallel quadruplex.[15] The CD spectra of the samples with the 3′ and 5′ tails are characteristic of the anti-parallel chair type structure as shown in File S2. The CD spectra of the samples with the 3′ and 5′ tails are nearly identical. The lack of change in the CD pattern and the similarity in the cleavage patterns of the samples with 3′ and 5′ tails indicate that the presence of the tails has little effect on the structure of the 15 mer.

### Cleavage Patterns of Complexes of TBA with Drug like Molecules

The cleavage patterns were obtained as a function of the concentration for the four ligands. NMM has been shown to have high specificity for quadruplex structures over other forms of DNA and its fluorescence increases significantly in the presence of quadruplex DNA.[12], [50] TmPyP4 has been shown to bind to quadruplex DNAs with a large increase in fluorescence.[12] TmPyP4 binding to c-myc and other quadruplexes has been used to gain information about the effects of ligand binding on promoter strength and other effects.[46], [51], [52] There is a crystal structure of a complex of TmPyP4 with a quadruplex DNA [47] as well as an NMR structure of a complex with a different quadruplex DNA.[53] NSC 176319 was found to bind to quadruplex DNA by screening.[9] A structural similarity search based on NSC 176319 identified NSC 91881 and quadruplex binding was identified by our screening assay. The structures of these four are shown in Figure 1.

**Figure 3:**
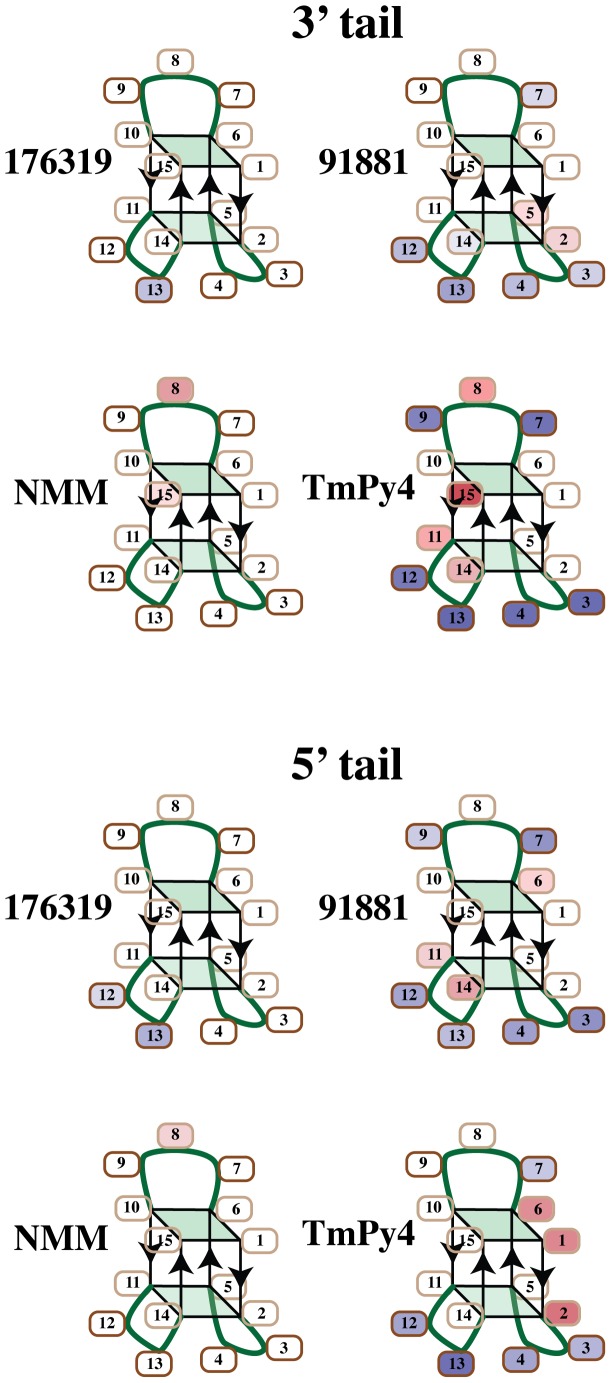
Depiction of the changes in cleavage induced by the four drug like molecules. The changes in cleavage of the residues are depicted using the chair type quadruplex structure of the DNA with blue indicating protection and red indicating enhanced cleavage. The changes for the 5′ and 3′ tail samples are both shown. Darker colors indicate a larger percentage change in the extent of cleavage.

For the cleavage experiments the DNA was annealed in 100 mM NaCl, 10 mM KCl buffer to obtain the chair conformer. The drug like molecule was added to the annealed DNA sample. The cleavage reactions were then carried out and the DNA cleavage fragments purified. The cleavage in the presence of the drug like molecule was quantified and the results are displayed as percent change in cleavage in Figure 3. The cleavage results are also tabulated in File S4 for all of the concentrations examined.

NSC 176319 appears to primarily alter the extent of cleavage of residues 11, 12, 13 and 14 as depicted in Figure 3. The largest change is at residue 13 with more than 30% protection at the highest concentration examined. These four residues are spatially close together in the chair structure. These results are consistent with NSC 176319 binding to a single site as discussed below. There is also some change in the cleavage at T9 that may be due to partial binding at a second site.

The results indicate that NSC 91881 binding changes the extent of cleavage of the loop dT residues as well as some of the dG residues in the quartets. A single NSC 91881 is not nearly large enough to interact with all of these residues. A single NSC 91881 could interact with the dT residues 3, 4, 12, 13 since these two loops are spatially close together. Another binding site could be with the top loop. It appears that NSC 91881 binds to two or more sites of the 15 mer under these conditions as confirmed by the NMR results discussed below.

The cleavage at residue G8 in the TGT loop is preferentially altered by the addition of NMM. The binding could be to the top quartet formed by residues 1, 6, 10, 15 or to the TGT loop. We have previously shown that NMM binding does not significantly change the CD spectrum of the 15 mer.[15] The CD result indicate that NMM binding does not alter the folding pattern of this DNA and this is consistent with the cleavage results that indicate localized rather than widespread changes in cleavage.

The cleavage results with TmPyP4 are also depicted in Figure 3. The cleavage pattern indicates that TmPyP4 binds to multiple sites of the DNA as changes in cleavage are observed for most of the residues. The changes in the extent of cleavage as a function of TmPyP4 concentration indicate that the binding constants of these sites are similar. At TmPyP4 concentrations above about 10 µM there are very large changes in cleavage. An investigation of the TmPyP4 binding to a human telomeric quadruplex found that there were up to four binding sites per quadruplex.[54] The results on TmPyP4 binding to TBA also indicate multiple binding sites.

These cleavage results indicate that NSC 176319 and NMM each have a preferential binding site while NSC 91881 and TmPyP4 bind to multiple sites. The effect of NMM appears to be primarily on a single residue and there are many ways that the NMM complex could form leading to a selective effect on this single residue. NSC 176319 appears to alter the cleavage at four residues that are spatially close together in the TT loops of the chair structure.

### Comparison of Cleavage Results with Prior NMR, Crystallography and other Data

Complexes of TmPyP4 with quadruplex DNA have been determined by crystallography and by NMR [47], [53] and the two structures are rather different.[7] Studies of the binding of TmPyP4 have indicated that it binds to a wide range of DNA structural types [50] and has multiple binding modes.[47] The cleavage results indicate that TmPyP4 binds to multiple sites of the 15 mer. Taken together the results indicate that TmPyP4 has low site specificity and this lowers the interest in unraveling the details of the multiple binding sites as well as using TmPyP4 as a lead molecule for quadruplex specific binders.

NMM has been shown to have high selectivity towards quadruplex structures.[12], [50] This has led to many studies of the interactions on NMM with quadruplex DNA including the use of immobilized NMM to purify quadruplex DNA [55] and examination of the interactions with telomere DNA.[8] Sometime ago we attempted to obtain NMR data on complexes of NMM with quadruplex DNAs but solubility problems did not allow this.[56] The hydroxyl radical cleavage results indicate that NMM appears to preferentially change the cleavage of the central dG residue of the TGT loop.

NSC 176319 and NSC 91881 are structurally distinct from the molecules that have been investigated in complexes with quadruplex DNA by crystallography and NMR. A recent NMR based study found that a modified quindoline bound to a c-myc quadruplex at a quartet-loop junction interacting with two dG residues in a quartet as well as loop residues [13].

### NMR Results on the Complex of NSC 176319 with TBA

The hydroxyl radical cleavage results indicate that NSC 176319 binds primarily to the two TT loops of TBA. The NMR structures of TBA have T4 and T13 hydrogen bonding to each other with these residues stacked on the G2-G5-G10-G14 quartet.[31], [32], [38]–[40], [49] With the sample at 288 K the resonances of the imino protons of T4, T13, T8 and T9 and of the quartets can be observed.[31], [32], [38]–[40], [49] In addition, the methyl resonances of T4 and T13 are the most upfield of TBA.[31], [32], [38]–[40], [49] If NSC 176319 preferentially binds to the two TT loops then the prediction is that the imino and methyl resonances of T4 and T13 should be selectively altered by the complex formation.

**Figure 4:**
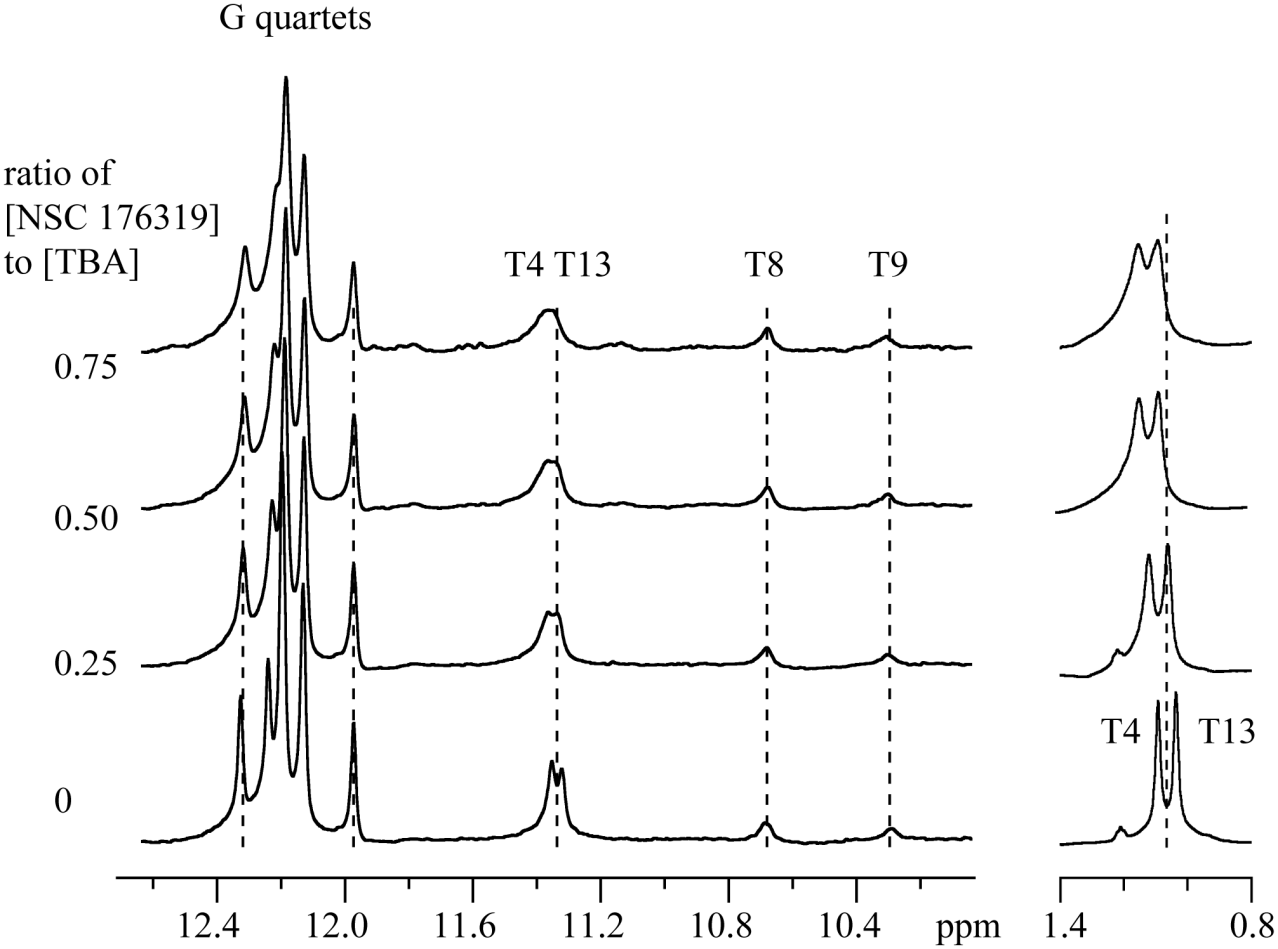
The 600 MHz spectra of TBA in the presence and absence of NSC 176319. The samples were at 288 K with the TBA at 1 mM. The integrated intensity of the G quartet region is the same in each spectrum. The dashed lines are to indicate which resonances change position as a function of NSC 176319 concentration.

**Figure 5:**
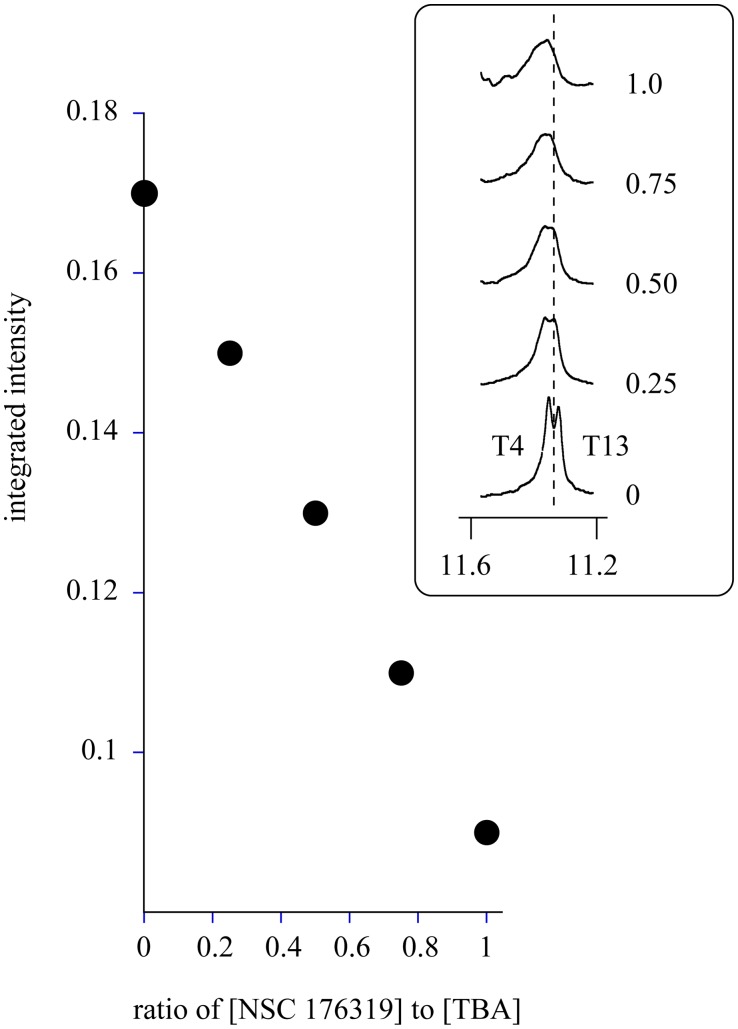
Effects of NSC 176319 on the intensities of the imino resonances of T4 and T13. The ratio of the integrated intensity of the T4 and T13 resonances to that of the G quartets is plotted as a function of NSC 176319 concentration. The region of the 600

The imino region of the spectrum of TBA is shown in figure 4 as a function of the concentration of NSC 176319. The results show that the presence of NSC 176319 leads to a loss of intensity from the T4 and T13 imino protons as well as a small downfield shift in the resonances. The plot of the integrated intensity of the T4 and T13 imino resonances shown in figure 5 indicates a linear decrease as a function of the concentration of NSC 176319. These results indicate that the binding of NSC 176319 disrupts the hydrogen bonding between T4 and T13.

Additional information comes from the methyl resonances of T4 and T13 as shown in figure 4. Both of these resonances move downfield upon addition of NSC 176319. This change is consistent with a destacking of these two residues from the G2-G5-G10-G14 quartet leading to smaller ring current shifts. Thus, the NMR and hydroxyl radical cleavage results are consistent with NSC 176319 binding causing a disruption of the T4–T13 hydrogen bonding and the destacking these residues from the bottom quartet. Modeling of the complex will need to take into consideration this structural change.

### NMR Results on the Complex of NSC 91881 with TBA

The NMR data on this complex indicated broadening of resonances from the top loop, the quartets and the TT loops. This data is consistent with binding to multiple sites. The presence of multiple binding sites is consistent with the cleavage results. Representative NMR data on this complex are included in File S5.

### Summary

The results of the enhanced hydroxyl radical cleavage protocol show that NSC 176319 and NMM appear to preferentially bind to single sites of the chair type quadruplex structure formed by TBA while TmPyP4 and NSC 91881 appear to bind to multiple sites. NMR methods have been used to show that NSC 176319 selectively disrupts the hydrogen bonding between residues T4 and T13 as well as shifting the methyl resonances of these residues downfield indicating destacking of T4 and T13 from the bottom quartet. NMR results on complexes of NSC 91881 indicated the presence of multiple binding sites. The NMR results in both cases are in excellent agreement with the hydroxyl radical cleavage results and offer validation of the approach. This methodology is now being applied to quadruplex DNAs based on human sequences and additional analogues of NSC 176319 are being examined.

## Supporting Information

File S1 The predicted cleavage and that observed for the 3′ and 5′ tail samples is presented in table and histogram format.
(DOCX)

File S2 The CD spectra of TBA and of the 3′ and 5′ tail samples.
(DOCX)

File S3 The normalization procedure for the gel data and an example.
(DOCX)

File S4 Procedures for tabulation of the cleavage results for the 3′ and 5′ tail samples in the presence and absence of the four drug like molecules.
(DOCX)

File S5 NMR spectra of TBA in the presence and absence of NSC 91881.
(DOCX)
