# Supplementary material for: Finding and Characterizing the Complexes of Drug Like Molecules with Quadruplex DNA: Combined Use of an Enhanced Hydroxyl Radical Cleavage Protocol and NMR
Source: PLoS One. 2014 Apr 24;9(4):e96218. doi: 10.1371/journal.pone.0096218 (PMC3999192; doi:10.1371/journal.pone.0096218)

**Supporting Information**

***S1.***

*Normalized observed and predicted cleavage values.*

Values for the 3’ and 5’ tail samples are from the experimental data and those for the 1C32 and 148D structures (taken from the Protein Data Bank) were calculated as described in the text. The results are also presented in the histogram.

|  | 1C32 | 148D | 5’ tail | 3’ tail |
| --- | --- | --- | --- | --- |
| G1 | 100 | 92 | 75 |  |
| G2 | 38 | 22 | 71 | 63 |
| T3 | 70 | 59 | 100 | 85 |
| T4 | 29 | 20 | 72 | 53 |
| G5 | 25 | 44 | 64 | 54 |
| G6 | 28 | 17 | 62 | 60 |
| T7 | 28 | 58 | 97 | 95 |
| G8 | 27 | 20 | 64 | 53 |
| T9 | 55 | 40 | 63 | 49 |
| G10 | 46 | 15 | 65 | 58 |
| G11 | 12 | 17 | 63 | 51 |
| T12 | 77 | 100 | 89 | 100 |
| T13 | 28 | 16 | 69 | 57 |
| G14 | 32 | 35 | 96 | 50 |
| G15 | 52 | 59 |  | 57 |


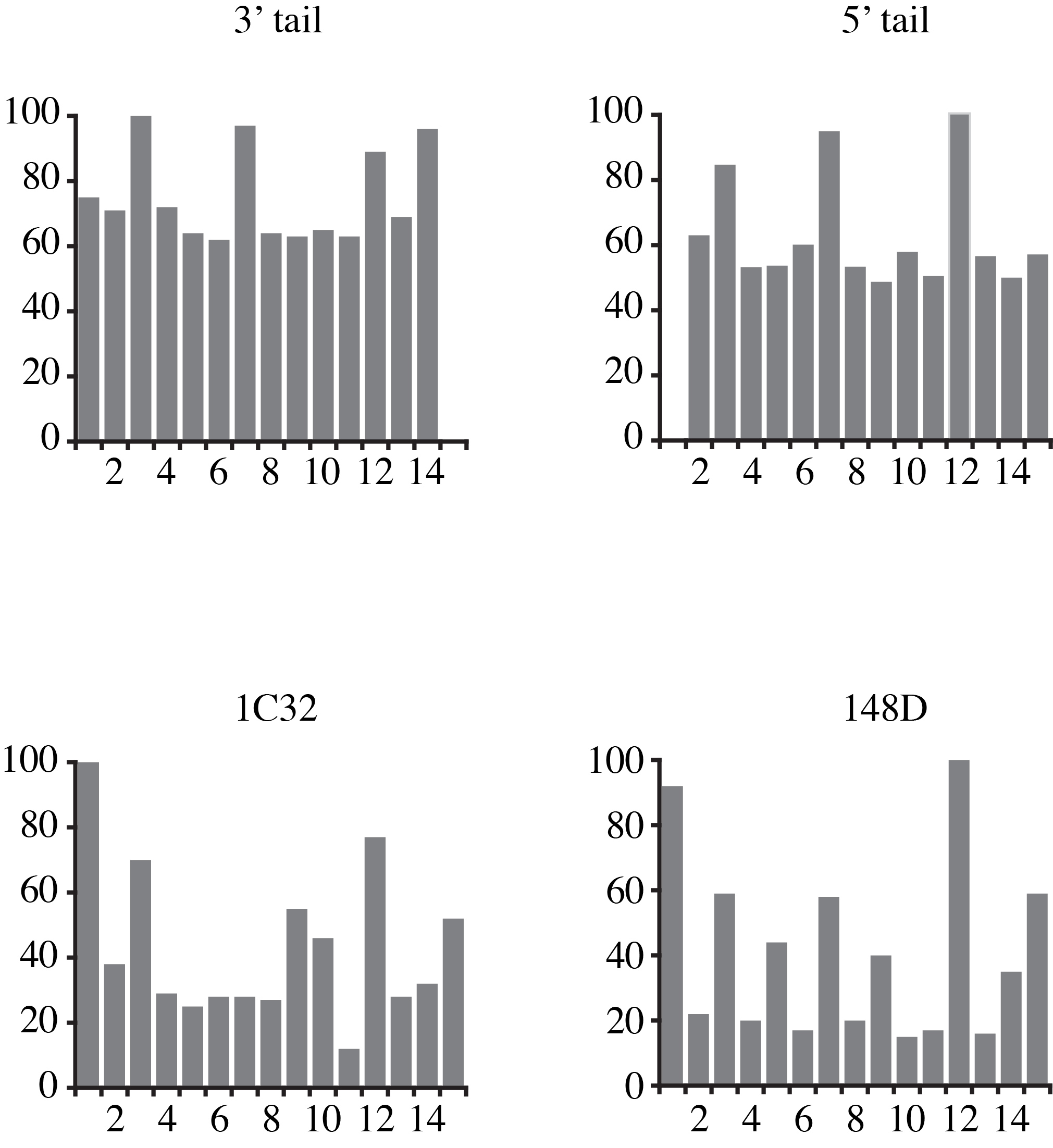

Supplement: File S1 — The predicted cleavage and that observed for the 3′ and 5′ tail samples is presented in table and histogram format. (DOCX) [file pone.0096218.s001.docx]
