# Supplementary material for: Finding and Characterizing the Complexes of Drug Like Molecules with Quadruplex DNA: Combined Use of an Enhanced Hydroxyl Radical Cleavage Protocol and NMR
Source: PLoS One. 2014 Apr 24;9(4):e96218. doi: 10.1371/journal.pone.0096218 (PMC3999192; doi:10.1371/journal.pone.0096218)

***File S2.***

*Circular dichroism spectroscopy.*

The CD experiments were performed using a Jasco J-810 spectropolarimeter equipped with a six cell holder with a programmable Peltier junction temperature controller. In the CD experiments the DNA was 10 μM in 2.5 mM Na_2_HPO_4,_ 10 mM KCl and 100 mM NaCl at pH 7, 293 K. The samples were annealed by heating at 263 K for 8 minutes and then gradually cooled to room temperature. Spectra were recorded in 0.2 cm quartz cuvette. The CD spectra were averaged over three scans using a 1 nm slit width from 200 to 320 nm at 0.1 nm intervals with a scan rate of 100 nM/min. Each spectrum was smoothed and the buffer spectrum subtracted using Spectra Analysis 2.04.02 software of the Jasco-810 Spectra Manager suite. The spectra are the same as for the DNA without a tail as reported previously.([1](#_ENREF_1))

1. Paramasivan, S., Rujan, I. and Bolton, P.H. (2007) Circular dichroism of quadruplex DNAs: applications to structure, cation effects and ligand binding. *Methods*, **43**, 324-331.


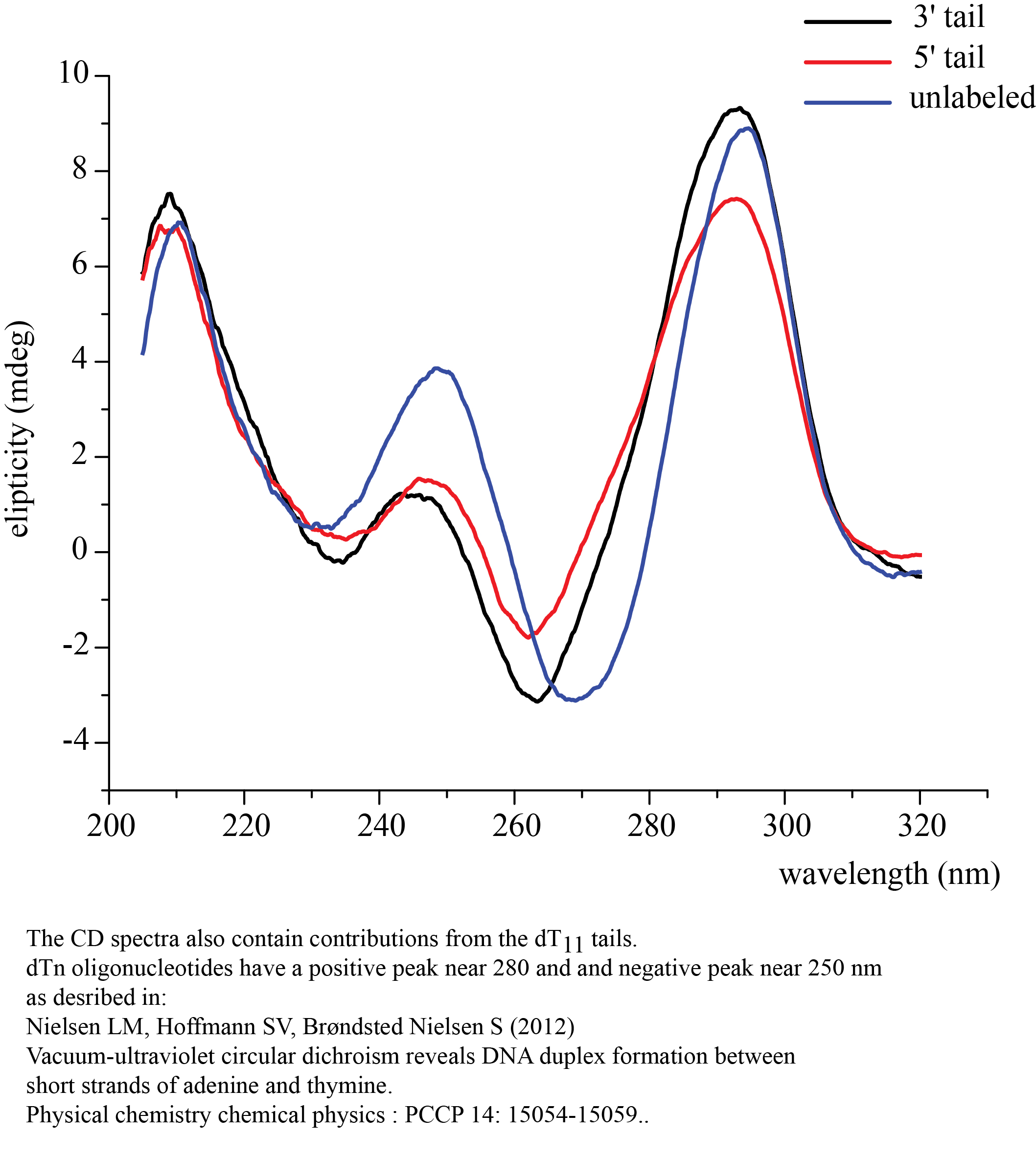

Supplement: File S2 — The CD spectra of TBA and of the 3′ and 5′ tail samples. (DOCX) [file pone.0096218.s002.docx]
