# Supplementary material for: Finding and Characterizing the Complexes of Drug Like Molecules with Quadruplex DNA: Combined Use of an Enhanced Hydroxyl Radical Cleavage Protocol and NMR
Source: PLoS One. 2014 Apr 24;9(4):e96218. doi: 10.1371/journal.pone.0096218 (PMC3999192; doi:10.1371/journal.pone.0096218)

***S3.***

*Normalization of gel band intensities.*

The band intensities of each gel lane were quantified using Semi-Automated Footprinting Analysis software, SAFA, v1.1. The intensities were exported to Excel for analysis. The sum of the intensities of individual bands in each lane, excluding the parent band, was normalized to 100. The percentage change in the normalized intensity for each band was calculated by ((I_0_-I_L_)/I_0_))*100 where I_0_ is normalized intensity of the band in absence of ligand and I_L_ the normalized intensity of the band in presence of ligand. The initial results were examined and a second normalization procedure was run with the bands that changed more than 15% excluded from the normalization since the presence of the ligand could alter the total cleavage of the DNA. Typical results for NSC 91881 are shown.


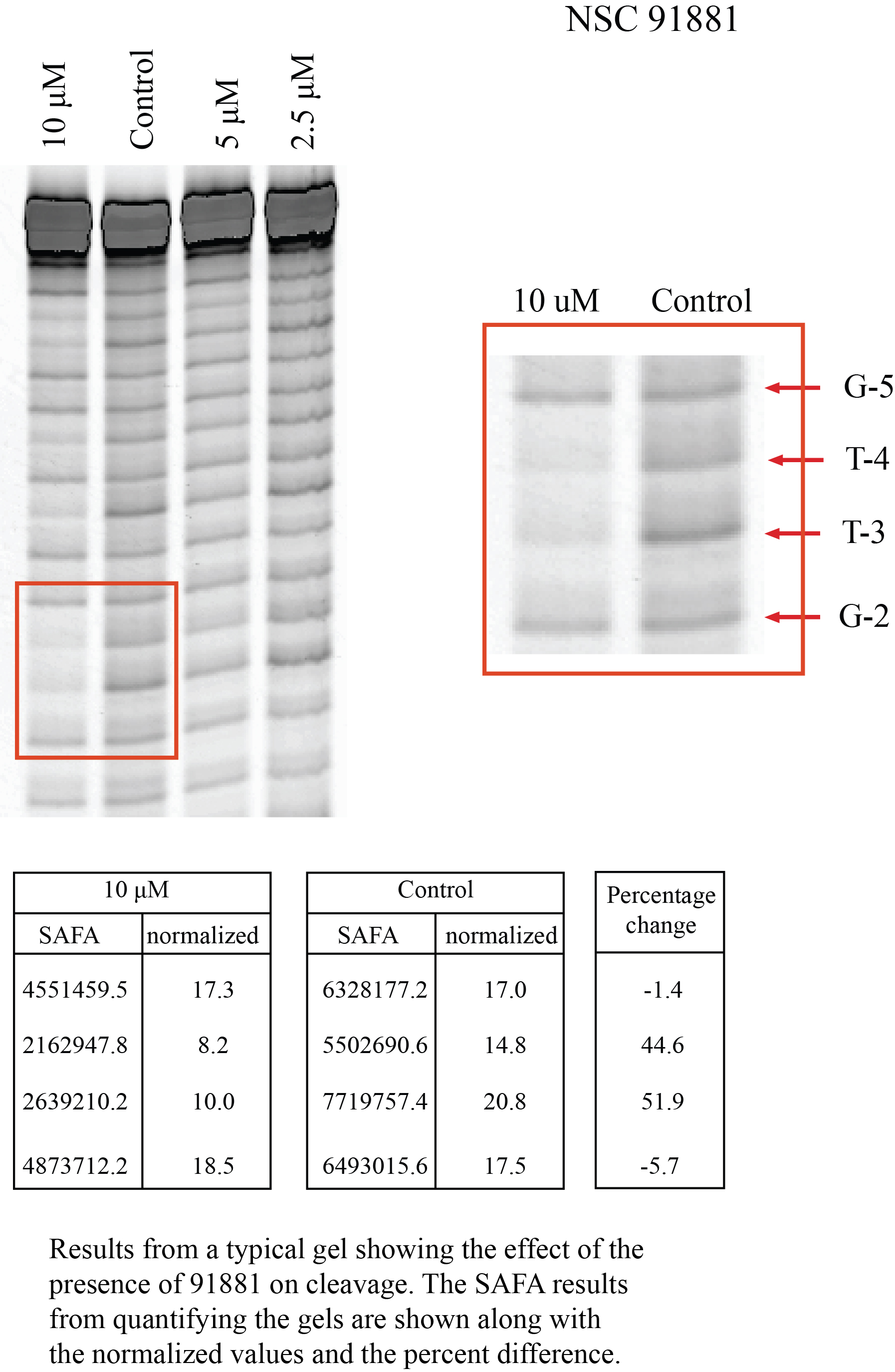

Supplement: File S3 — The normalization procedure for the gel data and an example. (DOCX) [file pone.0096218.s003.docx]
