# Supplementary material for: Finding and Characterizing the Complexes of Drug Like Molecules with Quadruplex DNA: Combined Use of an Enhanced Hydroxyl Radical Cleavage Protocol and NMR
Source: PLoS One. 2014 Apr 24;9(4):e96218. doi: 10.1371/journal.pone.0096218 (PMC3999192; doi:10.1371/journal.pone.0096218)
Supplement: File S4 — Procedures for tabulation of the cleavage results for the 3′ and 5′ tail samples in the presence and absence of the four drug like molecules. (DOCX) [file pone.0096218.s004.docx]

***S4.***

*Cleavage results for the 3’ and 5’ tail samples with the four drug like molecules.*

The reproducibility of the procedure was determined by running a cleavage reaction seven times. The analysis of the results indicated that the standard deviation in the intensity of any single band is on the order of 7% with the standard error of about 2% as described previously in reference [[38](#_ENREF_38)]. The results reported here are the averages of three runs.

3’ tail with NSC 176319

|  | 2.5 µM | 5 µM | 10 µM |
| --- | --- | --- | --- |
| G1 |  |  |  |
| G2 | -6.38 | -2.76 | -6.43 |
| T3 | 0.29 | 0.16 | 7.38 |
| T4 | -0.60 | 1.90 | 1.25 |
| G5 | 0.27 | -0.99 | -8.19 |
| G6 | -4.34 | -2.92 | -5.15 |
| T7 | 1.34 | -1.93 | -1.21 |
| G8 | 2.72 | 1.80 | -3.69 |
| T9 | -7.03 | -11.78 | -13.79 |
| G10 | 1.82 | -0.24 | -5.35 |
| G11 | 0.69 | -3.19 | -10.09 |
| T12 | -2.18 | -0.41 | 8.33 |
| T13 | 4.97 | 15.89 | 29.91 |
| G14 | 7.08 | 8.70 | 10.17 |
| G15 | 0.11 | 8.22 | 6.47 |

5’ tail, NSC with 176319

|  | 2.5 µM | 5 µM | 10 µM |
| --- | --- | --- | --- |
| G1 | 11.21 | 6.95 | -4.25 |
| G2 | 2.24 | 12.44 | -0.93 |
| T3 | -5.23 | -8.03 | 12.02 |
| T4 | -12.00 | -14.23 | -4.00 |
| G5 | -0.45 | 5.21 | -4.34 |
| G6 | 3.61 | 6.03 | -12.67 |
| T7 | -2.33 | -9.96 | 6.59 |
| G8 | -3.40 | 2.59 | -4.98 |
| T9 | 1.36 | -5.00 | -1.23 |
| G10 | -3.41 | 5.71 | -7.99 |
| G11 | 3.30 | 7.89 | -13.62 |
| T12 | 7.55 | 7.71 | 19.08 |
| T13 | 5.54 | 21.46 | 37.90 |
| G14 | -2.08 | 7.32 | -9.28 |
| G15 |  |  |  |

3’ tail with NMM

|  | 2.5 µM | 5 µM | 10 µM |
| --- | --- | --- | --- |
| G1 |  |  |  |
| G2 | 5.42 | 8.61 | 8.17 |
| T3 | 1.84 | 1.78 | 3.10 |
| T4 | -1.18 | 1.01 | 10.44 |
| G5 | 1.14 | 0.54 | 4.37 |
| G6 | -0.84 | -0.44 | -0.34 |
| T7 | 4.46 | 4.36 | 8.41 |
| G8 | -18.47 | -31.98 | -43.41 |
| T9 | -3.51 | -5.84 | -9.48 |
| G10 | 0.30 | 1.02 | 2.60 |
| G11 | -3.04 | -1.43 | -0.84 |
| T12 | 2.23 | 1.53 | -2.08 |
| T13 | -1.13 | -0.13 | -0.76 |
| G14 | -2.34 | -3.03 | -7.14 |
| G15 | -5.37 | -6.01 | -15.44 |

5’ tail with NMM

|  | 2.5 µM | 5 µM | 10 µM |
| --- | --- | --- | --- |
| G1 | -5.72 | -1.83 | -4.24 |
| G2 | -1.89 | -1.79 | -4.58 |
| T3 | -1.02 | -0.64 | 7.64 |
| T4 | 1.63 | 0.92 | 2.48 |
| G5 | -1.15 | -1.90 | -3.42 |
| G6 | 1.93 | -1.70 | -8.15 |
| T7 | 3.03 | 2.86 | 9.08 |
| G8 | -4.96 | -12.91 | -19.90 |
| T9 | -2.44 | -8.14 | -6.46 |
| G10 | 2.86 | -1.11 | -2.63 |
| G11 | 4.87 | 0.11 | -4.69 |
| T12 | 4.17 | 0.65 | 4.63 |
| T13 | -1.27 | 1.62 | 3.71 |
| G14 | -2.72 | 5.01 | 0.10 |
| G15 |  |  |  |

3’ tail with TMPyP4

|  |  | 2.5 µM | 5 µM | 10 µM |
| --- | --- | --- | --- | --- |
| G1 |  |  |  |  |
| G2 |  | -3.74 | 2.01 | -4.93 |
| T3 |  | 55.43 | 64.36 | 71.30 |
| T4 |  | 66.35 | 68.23 | 69.87 |
| G5 |  | -1.41 | -0.58 | 6.00 |
| G6 |  | -1.70 | -2.36 | -7.14 |
| T7 |  | 29.92 | 50.79 | 67.89 |
| G8 |  | -23.58 | -38.56 | -43.64 |
| T9 |  | 23.44 | 44.29 | 60.00 |
| G10 |  | 2.57 | 2.39 | 0.52 |
| G11 |  | -13.10 | -14.96 | -38.36 |
| T12 |  | 38.65 | 51.74 | 64.38 |
| T13 |  | 67.40 | 70.96 | 72.80 |
| G14 |  | -11.75 | -19.97 | -33.64 |
| G15 |  | -19.58 | -35.10 | -78.91 |

5’ tail with TMPyP4

|  | 2.5 µM | 5 µM | 10 µM |
| --- | --- | --- | --- |
| G1 | -10.15 | -18.69 | -52.99 |
| G2 | -13.86 | -28.13 | -61.81 |
| T3 | 40.92 | 46.16 | 35.50 |
| T4 | 40.22 | 37.60 | 43.28 |
| G5 | 7.02 | 2.66 | -8.40 |
| G6 | -10.52 | -20.84 | -49.92 |
| T7 | 12.01 | 26.95 | 27.31 |
| G8 | -12.15 | -4.72 | 0.72 |
| T9 | -4.48 | -4.93 | 3.31 |
| G10 | 4.95 | 6.39 | 12.16 |
| G11 | 4.49 | -0.31 | -7.18 |
| T12 | 25.38 | 32.65 | 44.22 |
| T13 | 41.96 | 47.38 | 68.55 |
| G14 | 18.47 | 16.11 | 14.81 |
| G15 |  |  |  |

3’ tail with NSC 91881

|  |  | 2.5 µM | 5 µM | 10 µM |
| --- | --- | --- | --- | --- |
| G1 |  |  |  |  |
| G2 |  | -6.64 | -1.46 | -19.36 |
| T3 |  | 3.12 | 11.08 | 22.56 |
| T4 |  | 10.27 | 21.36 | 31.58 |
| G5 |  | 3.86 | 1.98 | -17.16 |
| G6 |  | -17.82 | -14.99 | -9.14 |
| T7 |  | 9.29 | 10.72 | 19.43 |
| G8 |  | -6.50 | -6.42 | -9.18 |
| T9 |  | -5.36 | -12.03 | -0.55 |
| G10 |  | 3.74 | 1.47 | -2.19 |
| G11 |  | -12.69 | -10.15 | 0.18 |
| T12 |  | 9.94 | 15.63 | 33.00 |
| T13 |  | 20.71 | 35.02 | 45.45 |
| G14 |  | 12.74 | 11.10 | 11.41 |
| G15 |  | 7.04 | 6.45 | 10.06 |

5’ tail with NSC 91881

|  | 5 µM | 10 µM |
| --- | --- | --- |
| G1 | 9.36 | -0.12 |
| G2 | 10.17 | -5.18 |
| T3 | 19.45 | 52.61 |
| T4 | 14.99 | 45.53 |
| G5 | 0.08 | 1.34 |
| G6 | -7.43 | -19.72 |
| T7 | 23.53 | 52.29 |
| G8 | -7.17 | -5.24 |
| T9 | -0.34 | 25.37 |
| G10 | -4.94 | -5.97 |
| G11 | -11.83 | -20.66 |
| T12 | 13.23 | 43.49 |
| T13 | 10.00 | 32.21 |
| G14 | -27.70 | -39.72 |
| G15 |  |  |
