# Supplementary material for: Finding and Characterizing the Complexes of Drug Like Molecules with Quadruplex DNA: Combined Use of an Enhanced Hydroxyl Radical Cleavage Protocol and NMR
Source: PLoS One. 2014 Apr 24;9(4):e96218. doi: 10.1371/journal.pone.0096218 (PMC3999192; doi:10.1371/journal.pone.0096218)

***S5.***

*NMR data on the TBA-NSC 91881 complex.*

NMR spectra of TBA in the presence and absence of NSC 91881 are shown. The spectra are of the same regions shown in Figure 4 of the manuscript. The presence of NSC 91881 broadens the resonances in the quartet region as well as the imino of T9. In addition, changes in the chemical shifts of a number of the imino resonances are observed upon addition of NSC 91881.


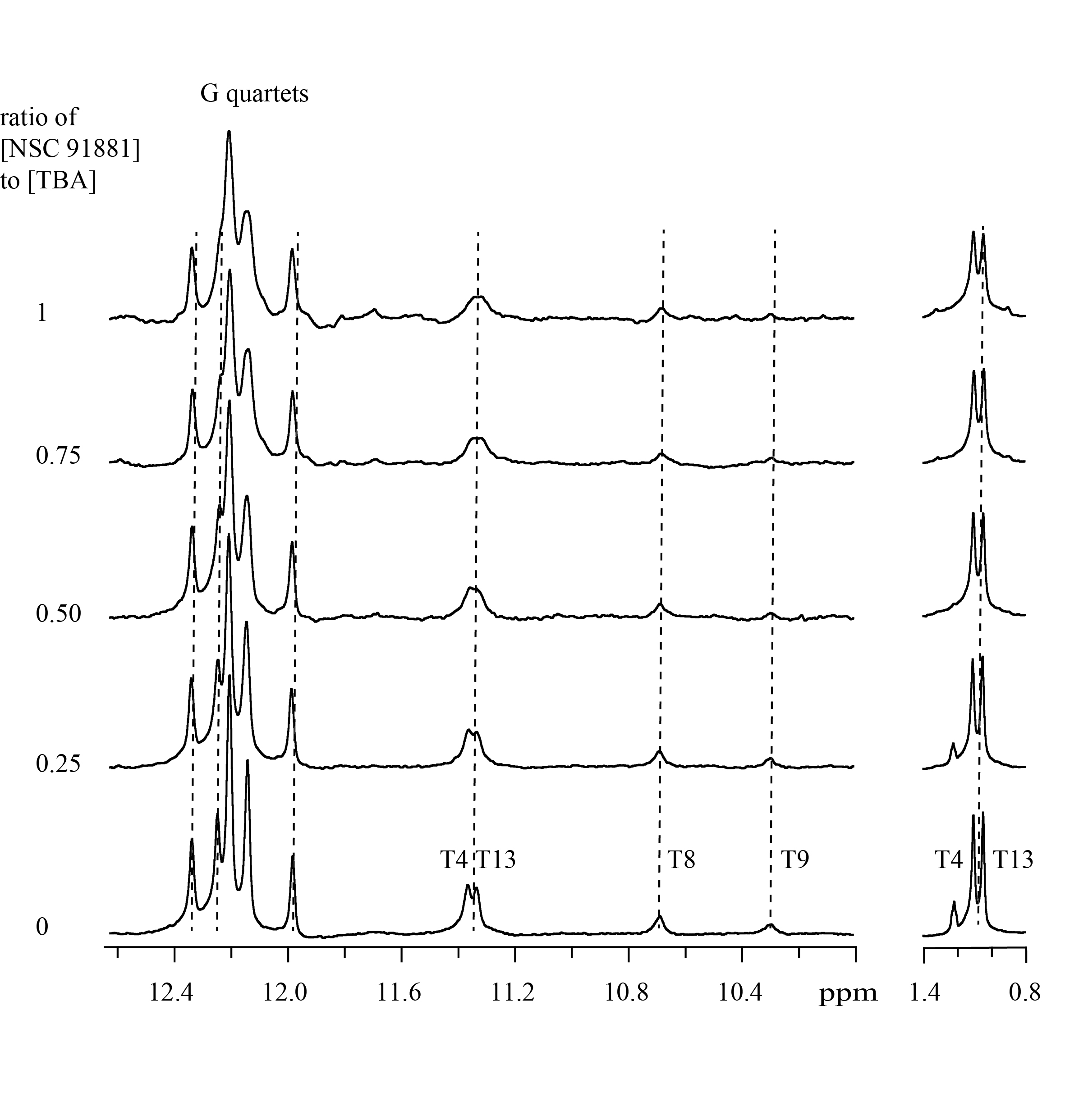

Supplement: File S5 — NMR spectra of TBA in the presence and absence of NSC 91881. (DOCX) [file pone.0096218.s005.docx]
